# Nimodipine augments cerebrovascular reactivity in aging but runs the risk of local perfusion reduction in acute cerebral ischemia

**DOI:** 10.3389/fnagi.2023.1175281

**Published:** 2023-04-27

**Authors:** Szilvia Kecskés, Ákos Menyhárt, Ferenc Bari, Eszter Farkas

**Affiliations:** ^1^Cerebral Blood Flow and Metabolism Research Group, Hungarian Centre of Excellence for Molecular Medicine – University of Szeged, Szeged, Hungary; ^2^Department of Cell Biology and Molecular Medicine, Albert Szent-Györgyi Medical School and Faculty of Science and Informatics, University of Szeged, Szeged, Hungary; ^3^Department of Medical Physics and Informatics, Albert Szent-Györgyi Medical School and Faculty of Science and Informatics, University of Szeged, Szeged, Hungary

**Keywords:** aging, cerebrovascular reactivity, hypercapnia, ischemia, Nimodipine

## Abstract

**Introduction:**

The efficacy of cerebrovascular reactivity (CVR) is taken as an indicator of cerebrovascular health.

**Methods and Results:**

We found that CVR tested with the inhalation of 10 % CO_2_ declined in the parietal cortex of 18-20-month-old rats. The CVR deficit in old rats was coincident with cerebrovascular smooth muscle cell and astrocyte senescence, revealed by the immuno-labeling of the cellular senescence marker p16 in these cells. In a next series of experiments, CVR was severely impaired in the acute phase of incomplete global forebrain ischemia produced by the bilateral occlusion of the common carotid arteries in young adult rats. In acute ischemia, CVR impairment often manifested as a perfusion drop rather than blood flow elevation in response to hypercapnia. Next, nimodipine, an L-type voltage-gated calcium channel antagonist was administered topically to rescue CVR in both aging, and cerebra ischemia. Nimodipine augmented CVR in the aged brain, but worsened CVR impairment in acute cerebral ischemia.

**Discussion:**

A careful evaluation of benefits and side effects of nimodipine is recommended, especially in acute ischemic stroke.

## 1. Introduction

Cerebrovascular reactivity (CVR) is a fundamental physiological process to match cerebral blood flow (CBF) to tissue demand. In essence, CVR reflects the capacity of cerebral blood vessels to dilate (or constrict) in response to a physiological stimulus and is an indicator of cerebrovascular reserve. The efficacy of CVR is evaluated, for example, by measuring the degree of vasodilation of an intracranial artery, or the increase of CBF in response to a hypercapnic challenge (i.e., CO_2_ inhalation or breath holding) (Fisher et al., 2018; Liu et al., 2019). There has been a growing interest in the clinical measurement of CVR, because CVR efficacy has been recognized as an indicator of cerebrovascular health (Juttukonda and Donahue, 2019). For example, CVR decline measured with BOLD MRI was related to internal carotid artery occlusion (De Vis et al., 2015). Further, reduced CVR corresponded with the severity of cerebral ischemia (Dettmers et al., 1993) and the risk for a future stroke attack (Reinhard et al., 2014; Papassin et al., 2021). Accumulating experimental (Mitschelen et al., 2009; Balbi et al., 2015) and clinical studies (Lu et al., 2011; Flück et al., 2014; Miller et al., 2019; Koep et al., 2022; Yew et al., 2022) also testify a link between CVR decline and healthy aging. In addition to revealing compromised cerebrovascular health, CVR deficit appears to correlate with weakening cognitive function in aging, carotid stenosis and dementia (Silvestrini et al., 2006; Mitschelen et al., 2009; Silvestrini et al., 2009; Lattanzi et al., 2018). The mechanistic and cellular background of altered CVR, however, remains inconclusive.

CVR hinges on the ability of cerebrovascular smooth muscle cells (SMCs) to dilate, as SMCs are the key elements in the vascular wall to execute vasodilation. In fact, CVR efficacy has been suggested to represent the functional integrity of cerebrovascular SMCs (Hayes et al., 2022). Deteriorating vascular SMC function is linked to cellular senescence, a chronic cellular stress response with a critical role in vascular aging (Minamino et al., 2004; Chi et al., 2019). In addition to the expression of cellular senescence markers including the protein p16, senescent vascular SMCs lose their ability to divide and their intracellular signaling cascades become altered (Chi et al., 2019). Despite the emerging evidence on the role of SMC senescence in vascular aging, it remains to be determined whether CVR impairment corresponds to cerebrovascular SMC senescence.

In addition to the postulated executive dysfunction of cerebrovascular SMCs with aging, the regulation of CVR is also prone to age-related deterioration. The tonic release of brain parenchyma-derived nitric oxide appears to be required as a permissive condition to hypercapnia-induced vasodilation (Iadecola, 1992; Brian Jr et al., 1996; Iadecola and Zhang, 1996), which is thought to become less effective with aging due to increased oxidative stress (Mayhan et al., 2008). On the other hand, the dynamic CBF increase in response to hypercapnia was shown to be mediated by cyclooxygenase-1 (COX-1)-derived prostanoids (Niwa et al., 2001), and COX-1 activation that produces vasodilator prostaglandin E_2_ (PGE_2_) to hypercapnia was linked to astrocytes (Howarth et al., 2017). Astrocyte senescence occurs with brain aging (Cohen and Torres, 2019), and seems to correlate with the dysfunction of neurovascular coupling (Yabluchanskiy et al., 2020), the mechanism to increase local perfusion in response to neural activity. Taken together, it is intriguing to explore whether astrocyte senescence is implicated in CVR impairment, as well. Finally, the involvement of endothelial cells in CVR is also plausible. Sophisticated genetic modification of mice recently revealed that CO_2_ induced cerebral vasodilation was in part also mediated via the endothelial proton-sensitive receptor GPR4, intracellular Gαq/11 proteins, and the release of prostacyclin and nitric oxide. The knock-out of the GPR4 receptor curbed the CO_2_ inhalation-induced CBF increase significantly, but the CBF increase still reached over 120%, and these data were obtained at a rather high concentration of inhaled CO_2_ (20%) (Wenzel et al., 2020).

Here we set out first to reproduce, in rodent experimental models, CVR deficit that progresses gradually with aging and has an abrupt onset after acute cerebral ischemia. Next, we labeled and quantified senescent cerebrovascular SMCs and astrocytes in aged animals to examine if senescence of these cellular elements coincides with age-related CVR dysfunction.

Building on these results, we further hypothesized that the pharmacological potentiation of vascular SMC relaxation may restore CVR impaired by age or ischemia. Cerebrovascular SMCs have recently been proposed as candidates for therapeutic purposes in neurodegenerative conditions (Hayes et al., 2022). Further, accepting the notion that the reduction of CVR may be a potential cause of the weakening of some cognitive abilities (Silvestrini et al., 2006; Mitschelen et al., 2009; Silvestrini et al., 2009; Lattanzi et al., 2018), the augmentation of CVR and cerebrovascular health emerges as a therapeutic target to improve neurologic function in aging and cerebral ischemia.

Nimodipine is an L-type voltage-gated calcium channel (VGCC) antagonist that targets vascular SMCs and is approved for the prevention of neurological deficits in subarachnoid hemorrhage (Carlson et al., 2020). There has been a renewed interest in finding additional indications and pharmaco-technologic solutions of drug delivery to exploit the benefits of nimodipine (Carlson et al., 2020; Tóth et al., 2020a). Nimodipine decreases cerebrovascular tone and increases CBF, but it is uncertain if the agent also augments cerebrovascular responses. We have recently found that nimodipine augments the CBF response to somatosensory stimulation after spreading depolarization in the otherwise intact mouse cerebral cortex (Menyhárt et al., 2023). Here we set out to investigate the possibility that nimodipine may rescue CVR efficacy impaired by aging and acute cerebral ischemia.

## 2. Materials and methods

Experimental procedures were approved by the National Food Chain Safety and Animal Health Directorate of Csongrád County, Hungary. The procedures were carried out according to the guidelines of the Scientific Committee of Animal Experimentation of the Hungarian Academy of Sciences (updated Law and Regulations on Animal Protection: 40/2013. (II. 14.) Gov. of Hungary), based on the EU Directive 2010/63/EU on the protection of animals used for scientific purposes. Experiments are reported in compliance with the ARRIVE guidelines (Kilkenny et al., 2010).

Young adult (2 months, 464 ± 50 g) and old (18–20 months, 772 ± 60 g) male Wistar rats (*n* = 41 before exclusions made, *n* = 23 after exclusions and used for full data analysis) were used. They were fed standard rodent food and had access to tap water *ad libitum*. All animals were reared under controlled conditions including temperature, humidity and daily light cycle (23°C, 12:12 h light/dark cycle, lighting at 7 am).

### 2.1. Surgical procedures

The preparation has followed previously published guidelines (Szabó et al., 2019; Varga et al., 2020; Szabó et al., 2021). Anesthesia was performed by inhalation of 2–1.5% isoflurane in a N_2_O:O_2_ (3:2) gas mixture using a head cone, while the animals breathed spontaneously. Body temperature was maintained throughout the surgery (37.2°C) by a biofeedback-controlled homoeothermic blanket (Harvard Apparatus, United States). Prior to surgery, animals were given atropine (0.1%, 0.01 mL, i.m.) in order to avoid the production of airway mucus. In addition, the left femoral artery was cannulated to measure arterial blood pressure using a microtip pressure catheter (Micro-Tip BP Foundation System, AD Instruments, Australia) and arterial blood samples were collected for arterial blood gas analysis.

To induce incomplete global forebrain ischemia, a midline ventral cervical incision was made under lidocaine (1%) analgesia after shaving the cervical region. The common carotid arteries were carefully separated from the surrounding nerves and connective tissue, and surgical thread was looped around both arteries to perform later permanent occlusion.

To create a cranial window for CBF measurement and topical drug administration, the animals were placed in a stereotaxic apparatus (Stoelting Co., Wood Dale, IL, United States). After retraction of the skin over the parietal and temporal bones, a 3 × 3 mm craniotomy was created over the right parietal cortex 3 mm caudal to bregma and 5 mm lateral to the sagittal suture, using an electric drill under saline cooling (ProLab Basic, Bien Air 810; Switzerland). The dura mater was incised, and the changes in CBF were assessed using laser-Doppler flowmetry (LDF). Artificial cerebrospinal fluid (aCSF; mM concentrations: 126.6 NaCl, 3 KCl, 1.5 CaCl_2_, 1.2 MgCl_2_, 24.5 NaHCO_3_, 6.7 urea, 3.7 glucose, 95% O_2_ and 5% CO_2_ in a bubble to achieve a constant pH of 7.4) was applied topically in the cranial window to keep the exposed tissue moist.

### 2.2. Registration of local cerebral blood flow

LDF was used to monitor local CBF changes in response to hypercapnia (Probe 403 connected to a PeriFlux 5,000; Perimed AB, Sweden). An LDF needle probe was placed over a region devoid of visible pial blood vessels and with a baseline perfusion around 300 perfusion units (PFU). CBF signals were digitized using a PowerLab data acquisition device controlled by a LabChart 8 software (ADInstruments, Australia) at a sampling frequency of 2 kHz.

### 2.3. Experimental protocol and pharmacological treatment

**Figure 1:**
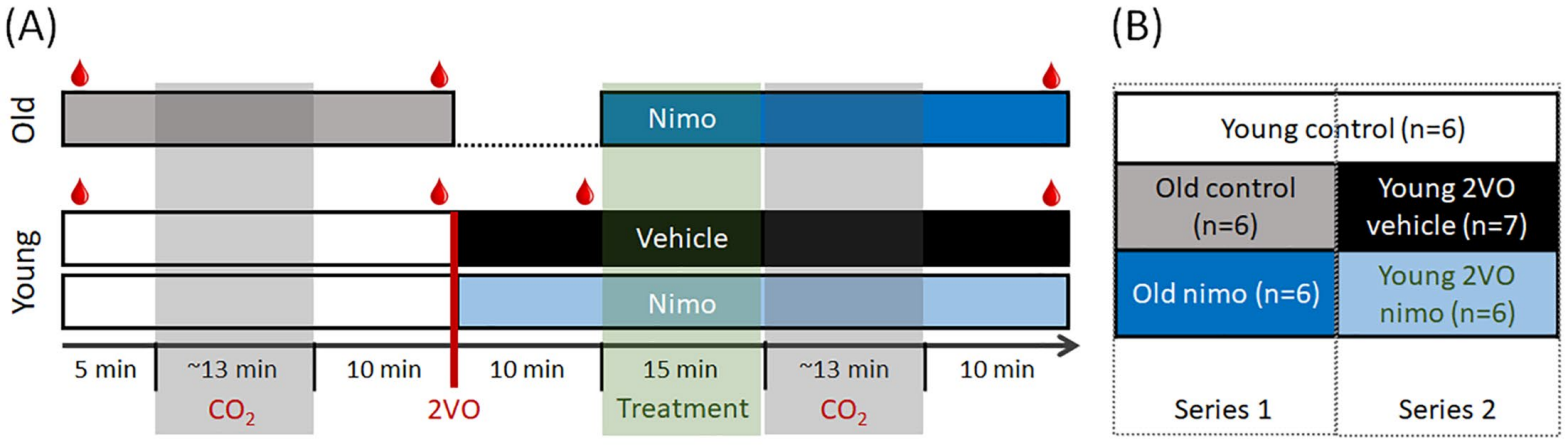
Experimental protocols **(A)** and experimental conditions compared **(B)**. For blood gas analysis, arterial blood samples were collected repeatedly (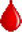 in A). Number of cases per group are given **(B)** after implementing the exclusion criteria. 2VO, bilateral common carotid artery occlusion; Nimo, nimodipine.

First, we set out to recapitulate the impact of aging or acute ischemia on CVR. Next, we explored whether nimodipine administration improved the age-related or the acute ischemia-induced impairment of CVR. To achieve these goals, we ran two series of experiments. In *Series 1*, aged animals were used. In *Series 2*, incomplete global forebrain ischemia was created in young animals (Figure 1).

The experimental protocol in both *Series* was initiated 30–60 min after the surgical preparation was completed. The efficacy of cerebrovascular reactivity was evaluated with a hypercapnic challenge. Brief episodes of 10% CO_2_ inhalation were imposed for 20 s, which was repeated 5 times at 3-min intervals in a block (Howarth et al., 2017). Corresponding changes in CBF were identified and recorded using LDF. Experiments were terminated with the overdose of isoflurane or with transcardiac perfusion in deep anesthesia.

*Series 1* involving old rats (*n* = 10 before exclusions made, *n* = 6 used for full analysis) followed a longitudinal design (Figure 1A, top). First, hypercapnic challenge was produced 5 min after baseline CBF recording to serve as control. Next, nimodipine (100 μM in 0.1% DMSO in aCSF) was applied topically within the cranial window at a previously established, effective dose of the agent (Richter et al., 2002; Szabó et al., 2019; Tóth et al., 2020b). Nimodipine is light-sensitive; to ensure maximum bioavailability, drug administration was performed twice at 5-min intervals over 15 min. Fifteen min after nimodipine administration, the hypercapnic challenge was repeated.

*Series 2* involving young rats (*n* = 25 before exclusions made, *n* = 13 used for full analysis) also followed a longitudinal experimental paradigm (Figure 1A, bottom). The first hypercapnic challenge was produced 5 min after baseline CBF recording, as in *Series 1* to serve as control. Ten minutes later, incomplete global forebrain ischemia was created by the bilateral occlusion of the common carotid arteries (2VO). Successful 2VO was confirmed by a sudden drop of CBF. Nimodipine (*n* = 13) or vehicle (0.1% DMSO in aCSF; *n* = 12) was administered as in *Series 1*, 10 min after ischemia induction. Fifteen min after nimodipine administration, the hypercapnic challenge was repeated.

The initial, control segment of the protocol conducted on young rats also served as age-matched control for the old control condition (Figure 1B). Altogether, five experimental conditions were evaluated and compared: Young intact untreated (Young control), Old intact untreated (Old control), Old intact treated (Old nimo), Young ischemic non-treated (Young 2VO vehicle) and Young ischemic treated (Young 2VO nimo) (Figure 1B).

Additional young rats (*n* = 6 before exclusions made, *n* = 4 finally used for histology) were sacrificed before 2VO induction, to be used for immunohistochemistry as young reference for the Old control group.

### 2.4. Immunohistochemistry

Four Young control and 4 Old control animals were subjected to transcardial perfusion under deep anesthesia. Perfusion with ice-cold physiological saline through the left cardiac ventricle was followed by 4% paraformaldehyde (PFA) solution. Brains were removed, stored in 4% PFA overnight, and then placed in 30% sucrose in phosphate buffered saline (PBS) for cryoprotection. Twenty-μm-thick coronal forebrain sections were made using a cryostat freezing microtome (Leica CM 1860 UV, Leica, Germany).

Sections were selected from three coronal planes according to the coordinates of the Paxinos and Watson atlas to investigate 6 specific brain areas: 1 mm rostral to bregma (cortex and striatum), 3 mm caudal to the bregma (dentate gyrus and hippocampus CA1 region) and 6 mm caudal to the bregma (caudal part of the dentate gyrus and caudal part of the hippocampus CA1 region); three sections were taken from each plane.

Cerebrovascular SMCs were labeled with alpha smooth muscle actin (αSMA) immunostaining (monoclonal primary anti-αSMA antibody produced in mouse, Genetex, U.S.A., GTX73419, 1 h). Astrocytes were identified by glial fibrillar acidic protein (GFAP) labeling (monoclonal primary anti-GFAP antibody produced in mouse, Sigma Aldrich, Hungary, G3893, 1:1500, 1 h). The senescent phenotype of cells was identified with p16 protein labeling (polyclonal primary anti-p16INK4a antibody produced in rabbit, Sigma Aldrich, Hungary, SAB5700620, 1:100, 1 h). The co-localization of the αSMA and p16 signals identified senescent cerebrovascular SMCs in cerebral arterioles, whereas the co-localization of the GFAP and p16 signals indicated senescent astrocytes. Non-specific protein binding sites were blocked by 10% normal goat serum (NGS) (Merck, Kenilworth, New Jersey, United States). Secondary antibodies included Goat anti-Mouse Alexa Fluor Plus 488 (Thermo Fisher, U.S.A., A32723, 1:100, 1 h) to label cerebrovascular SMCs or astrocytes with green, and Goat anti-Rabbit Alexa Fluor 555 (Thermo Fisher, U.S.A., A-21429, 1:100, 1 h) to label p16 with red fluorescent signal. Slices were mounted on slides and cover slipped with Fluoromount G (Thermo Fisher, U.S.A., 00–4,959-52) containing DAPI. Staining was visualized using a fluorescence microscope (Leica DM LB2, Leica Microsystems Wetzlar GmbH, Wetzlar Germany), microscopic images were captured with a LEICA DFC250 camera at 40x magnification (Leica Microsystems Wetzlar GmbH, Wetzlar Germany) and analyzed with ImageJ (National Institutes of Health, Bethesda, Maryland, United States) (Schneider et al., 2012).

### 2.5. Data processing and analysis

Data analysis was performed using AcqKnowledge 4.2 software (Biopac Systems, Inc., Goleta, USA). CBF traces were down-sampled to 1 Hz. Changes in CBF relative to baseline were expressed by using the average of the 5-min baseline considered 100% and the mean of the recorded biological zero as 0%.

Experiments for comprehensive data analysis were selected considering the following exclusion criteria. Animals with arterial blood pH below 7.3 during baseline were excluded from further analysis (*n* = 1 in *Series 1*, and *n* = 7 in *Series 2*). Intact cerebral autoregulation was confirmed by a correlation analysis between MABP and CBF over the recordings of each experimental animal (Menyhárt et al., 2022). Recordings indicating impaired cerebral autoregulation (correlation coefficient > 0.3) were excluded from further statistical analysis (*n* = 3 in *Series 1*, *n* = 5 in *Series 2*, and *n* = 2 among the young animals prepared for immunohistochemistry). Finally, detailed analysis of each event required an appropriate signal-to-noise ratio.

The efficacy of CVR was characterized by the relative and absolute peak amplitude, the rising slope, the latency, and the duration of the CBF response to hypercapnia. In the non-ischemic young and old conditions, the values of the five hypercapnic trials in a block were averaged and used as a single value per animal. Under ischemia, some of the trials triggered a negative, rather than a positive CBF deflection, and negative and positive CBF responses appeared random in a block. In such cases, the positive and negative CBF transients were evaluated separately. Manual cell counting of p16 and αSMA or GFAP immune-positive cells was performed by two independent investigators in the hemisphere contralateral to the cranial window, at 40x magnification in a unit area of 1 mm^2^. The examination was repeated in three slides per coronal plane per rat, and the results were averaged for each region in each animal.

Data are presented as mean ± standard deviation. Statistical analysis of data sets was performed using SigmaPlot (version 12.5, Jandel, Inc., United States) software. Normal distribution was confirmed with a Shapiro–Wilk test. Data sets with normal distribution were further evaluated with a paired T-test in case sampling points were related, or one-way analysis of variance (ANOVA) followed by a Holm Sidak *post hoc* test. In case of non-normal distribution of data, Kruskal-Wallis analysis was applied. For serial data, an RM ANOVA was used followed by the Holm Sidak *post hoc* test. Significance levels were defined as *p* < 0.05^*^, *p* < 0.01^**^ and *p* < 0.001^***^. Distinct statistical methods are provided in detail in each Figure legend.

## 3. Results

### 3.1. Physiological variables and the effect of nimodipine on MABP and CBF

Physiological variables were monitored repeatedly over the experimental protocol. Arterial blood gases were in the physiological range in all the animals included for comprehensive data analysis (before the hypercapnic challenge: pO_2_: 143.4 ± 6.5 mmHg, pCO_2_: 39.8 ± 6.5 mmHg, pH: 7.4 ± 0.1). MABP during baseline varied around 95.1 ± 8.7 mmHg in the young and 82.1 ± 13.3 mmHg in the old groups with no statistical difference. The onset of ischemia increased MABP notably from 98.7 ± 16.4 to 114.6 ± 18.5 mmHg in the young animals. CBF dropped down to 17.2 ± 13.2% shortly after ischemia onset and recovered to 27.8 ± 15.7% by the time CO_2_ inhalation was initiated.

**Figure 2:**
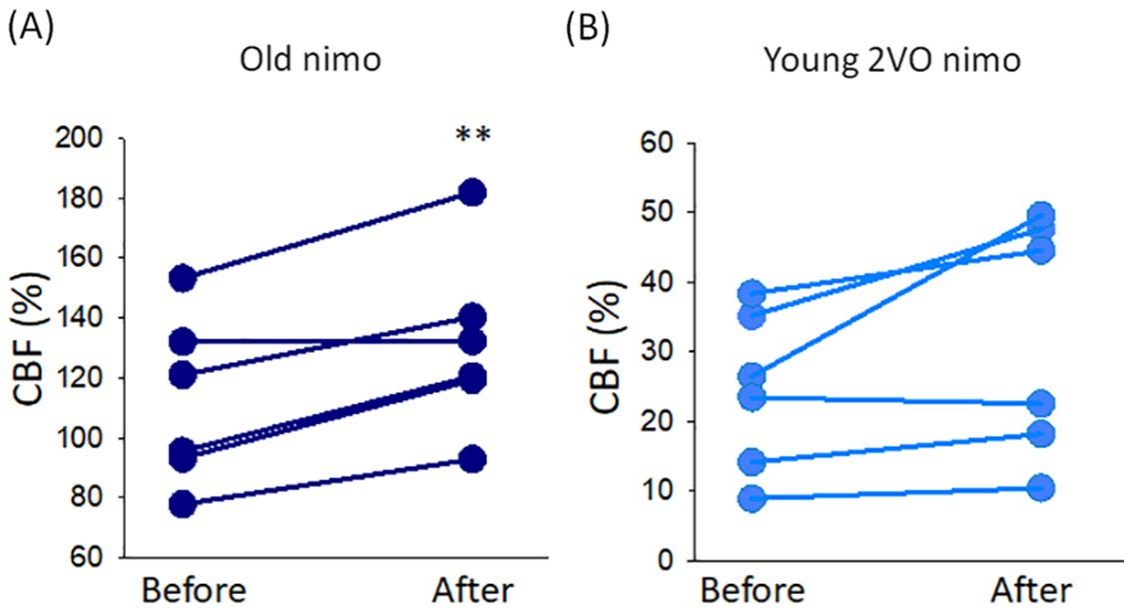
The impact of nimodipine treatment on cerebral blood flow (CBF). **(A)** Change in CBF in the Old nimo group shortly after nimodipine treatment (After) with respect to immediately prior to treatment (Before). **(B)** Change in CBF in the Young 2VO nimo group shortly after nimodipine treatment (After) with respect to immediately prior to treatment (Before). Data pairs for each animal are shown. For statistical analysis, the normal distribution of data was evaluated with a Shapiro–Wilk test (**A**: *p* = 0.234, **B**: *p* = 0.134), and then a paired *T*-test was used. The level of significance is labeled as *p* < 0.01 ^**^vs. before nimodipine treatment.

The potential impact of nimodipine on MABP and CBF was evaluated by comparing the values taken before and after nimodipine administration. CBF significantly increased after nimodipine application in old animals (136 ± 24 vs. 122 ± 26%, after vs. before in Old nimo) (Figure 2A). In the Young 2VO nimo group, CBF increase after nimodipine administration was obvious only in half of the animals, and CBF remained level in the rest of the group (Figure 2B). Taking the average, nimodipine did not alter CBF significantly in the Young 2VO nimo group (32 ± 17 vs. 24 ± 11%, after vs. before) (Figure 2B).

### 3.2. Cerebrovascular reactivity impaired by aging and acute cerebral ischemia

**Figure 3:**
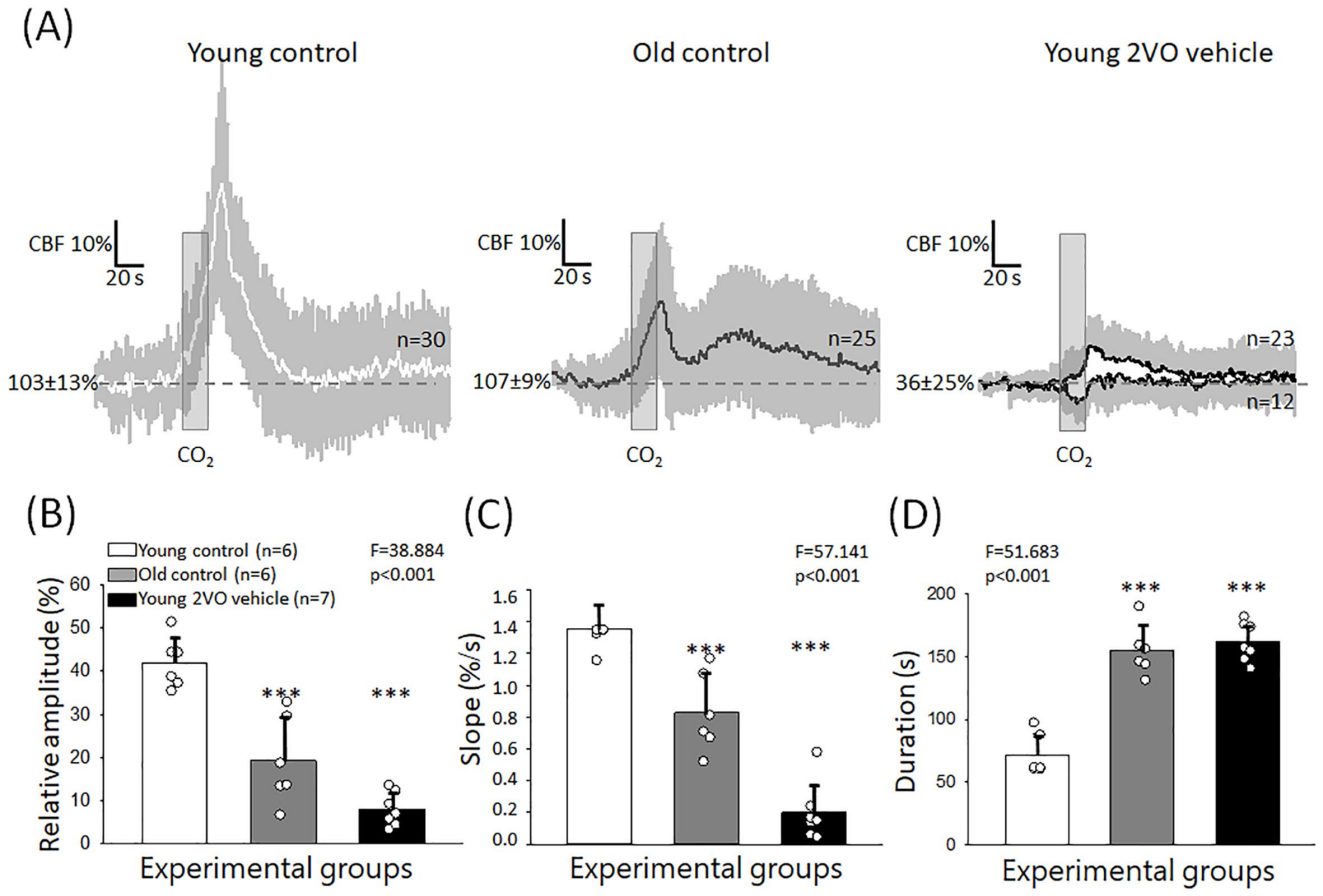
Hypercapnic vasodilation impaired by age and acute cerebral ischemia. **(A)** Mean traces (±stdev) of CBF changes with individual hypercapnic trials in the Young control, Old control and young acute cerebral ischemia (Young 2VO vehicle) groups. **(B)** The relative amplitude of hyperemia in response to hypercapnia. **(C)** The rate of hyperemia (rising slope). **(D)** The duration of hyperemia. In **B-D**, data are given as mean ± stdev. The normal distribution of data was evaluated with a Shapiro–Wilk test (**B**: *p* = 0.694, **C**: *p* = 0.058, **D**: *p* = 0.149). In **B–D**, one-way ANOVA was applied, followed by a Holm Sidak *post hoc* test. Levels of significance are shown as *p* < 0.001^***^ vs. Young control.

In *Series 1* (experiments on aging), transient CBF increase occurred invariably in response to hypercapnia in both young and old animals. However, the kinetics of the response changed notably with aging. In young rats, we consistently observed a monophasic, rapid, hyperemic response. In the old group, the response turned into bi-phasic, and the first, sharp peak was followed by a second, blunted peak (Figure 3A). Further, the impact of aging was characterized by the following variables. The relative amplitude of the first peak of the hyperemic response was significantly reduced in aged animals compared to the young group (19 ± 20 vs. 42 ± 12%, Old control vs. Young control) (Figure 3B). Similarly, the rate of hyperemia (rising slope) decreased (0.83 ± 0.49 vs. 1.36 ± 0.29%/s, Old control vs. Young control) (Figure 3C), while the duration of hyperemia increased with age (155 ± 40 vs. 72 ± 33 s, Old control vs. Young control) (Figure 3D).

In *Series 2* (experiments on ischemia), two distinct CVR types were observed after ischemia onset (Figure 3A). As described under Materials and Methods, a block of hypercapnic challenge consisted of 5 subsequent, brief (20 s) episodes of CO_2_ inhalation (Figure 1). In 2 animals, all 5 trials in a block were monophasic hyperemic with a single peak after 2VO. In 5 animals, however, a paradoxical, very rapid drop rather than a transient CBF increase was associated with some of the hypercapnic trials randomly interspersed with hyperemic response types in a block. Calculating the mean values, the amplitude of CVR greatly decreased under ischemic conditions (8 ± 8 vs. 42 ± 12%, Young 2VO vehicle vs. Young control) (Figure 3B). CVR dysregulation was also reflected in a lower rate of hyperemia (0.19 ± 0.38 vs. 1.36 ± 0.29%/s, Young 2VO vehicle vs. Young control) (Figure 3C) and in the increased duration of hyperemia (162 ± 32 vs. 72 ± 33 s, Young 2VO vehicle vs. Young control) (Figure 3D).

### 3.3. Immunohistochemical study

**Figure 4:**
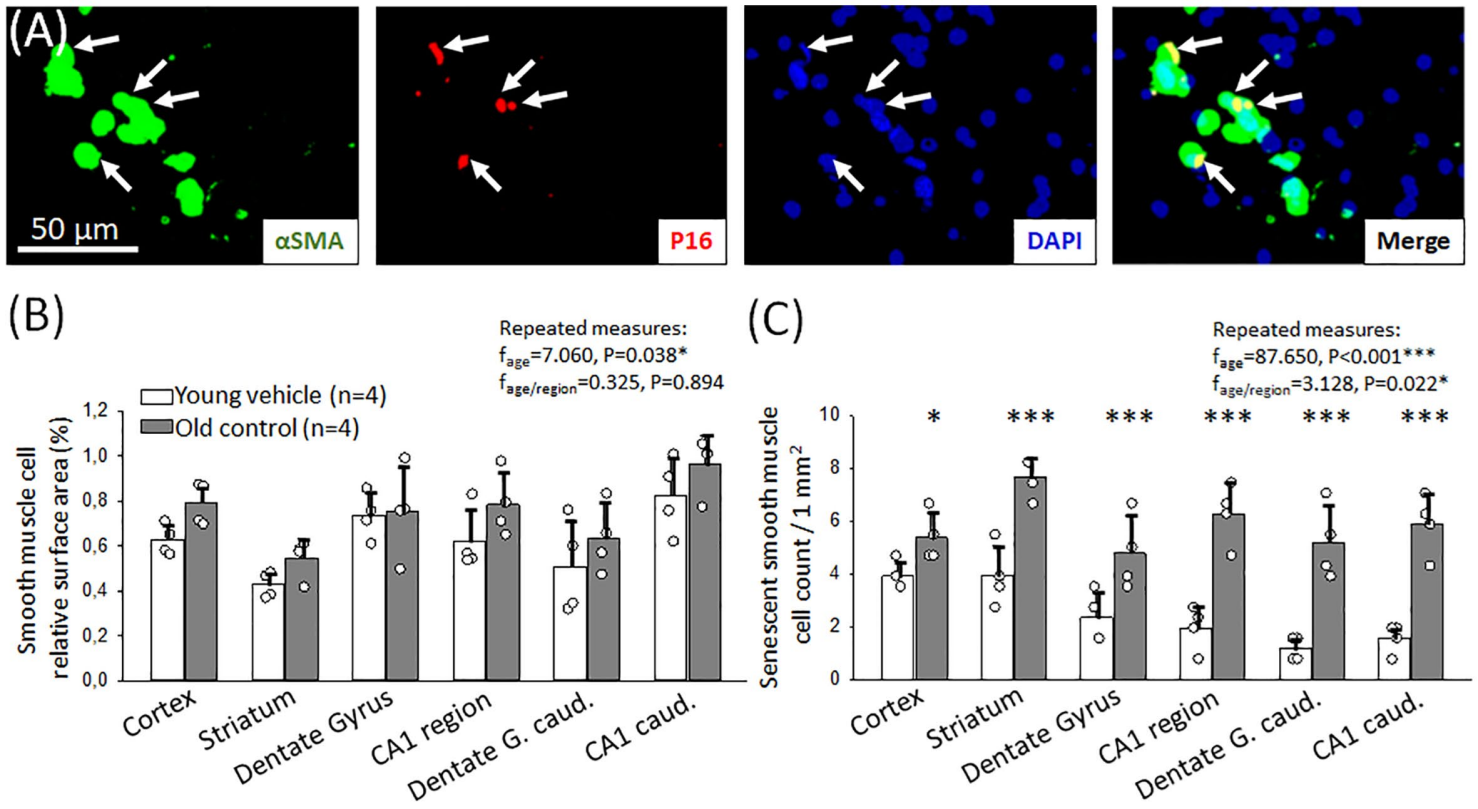
Immunocytochemical characterization of cerebrovascular smooth muscle cell (cerebrovascular SMC) senescence. **(A)** Representative fluorescence micrographs of αSMA and p16 co-localization (arrows) in the hippocampal CA1 region of an old animal. αSMA corresponding to cerebrovascular SMC coverage is shown in green, p16 labeling indicating senescent cells is red; and DAPI illustrates the cell nuclei in blue. **(B)** Relative area covered by cerebrovascular SMC labeled with αSMA in different forebrain regions in young and old animals. **(C)** The number of senescent cerebrovascular SMCs in young and old animals in different brain regions. Individual data points represent the mean value of three sections in each animal. Bar charts show mean ± stdev. The normal distribution of data was evaluated with a Shapiro–Wilk test (**B**: *p* = 0.488, **C**: *p* = 0.709). For further statistical analysis, a repeated measures model was used (RM ANOVA), followed by a Holm Sidak *post hoc* test. Levels of significance were set as *p* < 0.05^*^, *p* < 0.01^**^, *p* < 0.001^***^ vs. Young vehicle.

With age, the hyperemic response to hypercapnia deteriorated and thus CVR decreased in our experiments. This may be due to the senescence of cerebrovascular smooth muscle cells mediating hypercapnic vasodilation. To explore this hypothesis, we labeled cerebrovascular SMCs immunohistochemically with αSMA in old and young animals, and colocalized the cellular senescence marker p16 with αSMA (Figure 4A).

The relative surface area covered by αSMA-positive cerebrovascular SMCs was similar in both young and old rats (e.g., somatosensory cortex: 0.8 ± 0.1 vs. 0.6 ± 0.1%, Old control vs. Young vehicle) (Figure 4B). Co-localization of αSMA and the cellular senescence marker p16 signals identified senescent cerebrovascular SMCs. In old animals, a significant increase in the number of senescent cerebrovascular SMCs was found compared to young animals in all brain regions examined (Figure 4C). The most relevant increase was observed in the hippocampal CA1 region (3 mm to bregma: 6.3 ± 1.2 vs. 2.0 ± 0.8; 6 mm to bregma: 5.9 ± 1.2 vs. 1.6 ± 0.6 p16+ cerebrovascular SMCs/mm^2^, Old control vs. Young vehicle) (Figure 4C), as well as in the striatum and the dentate gyrus (striatum: 7.6 ± 0.8 vs. 3.9 ± 1.2; 3 mm to bregma: 4.8 ± 1.4 vs. 2.4 ± 0.9; 6 mm to bregma: 5.2 ± 1.4 vs. 1.2 ± 0.5 p16+ cerebrovascular SMCs/mm^2^, Old control vs. Young vehicle). An increased number of senescent cerebrovascular SMCs was counted in the cortex, as well (5.4 ± 0.9 vs. 3.9 ± 0.6 p16+ cerebrovascular SMCs/mm^2^, Old control vs. Young vehicle).

Astrocytes may also play an important mechanistic role in hypercapnic vasodilation (Grant et al., 2007; Howarth et al., 2017), therefore we investigated the senescence of astrocytes in aged animals compared to their young counterparts by the co-localization of p16 with GFAP.

**Figure 5:**
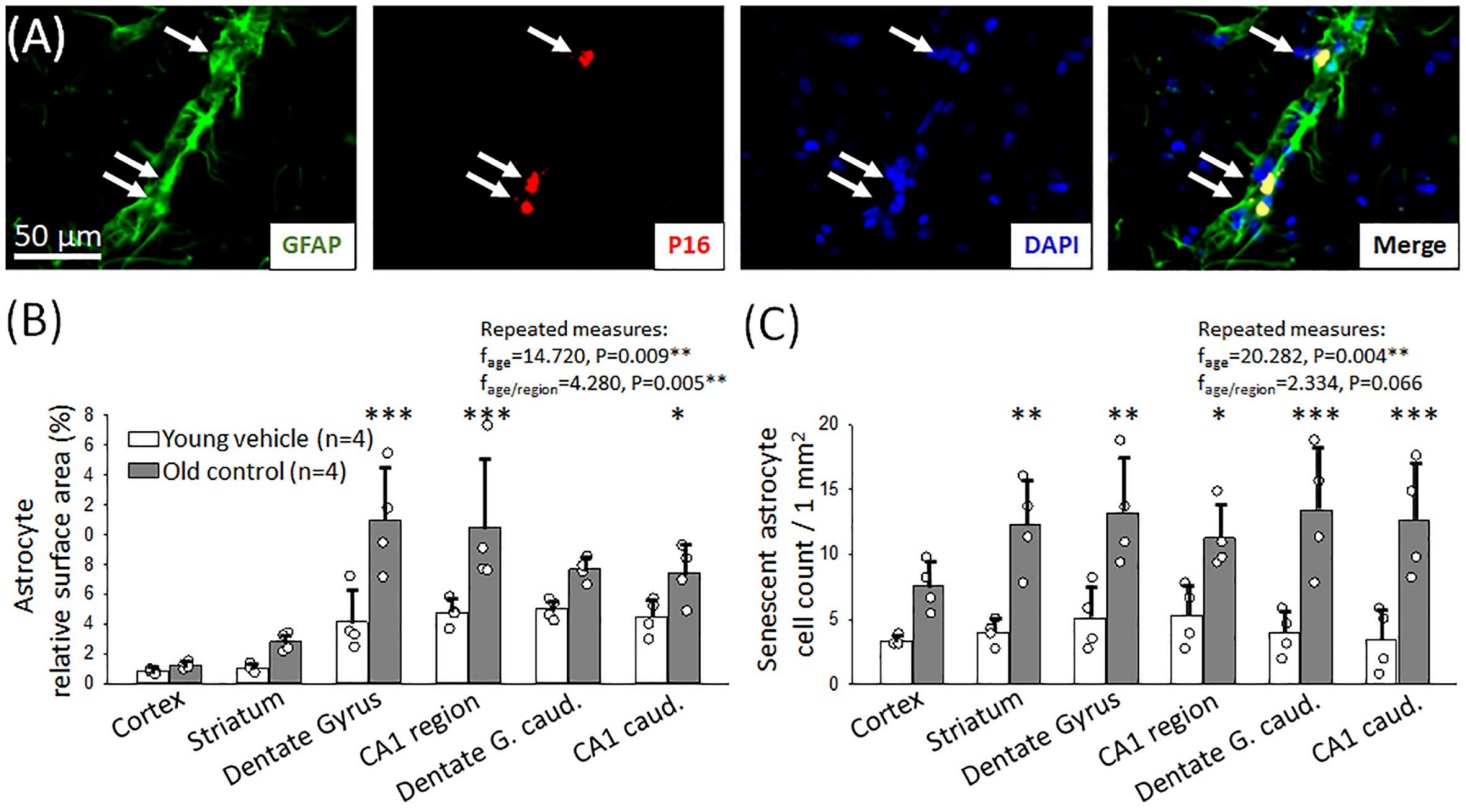
Immunocytochemical characterization of astrocyte senescence. **(A)** Representative fluorescence micrographs of GFAP and p16 co-localization (arrows) in the striatum region of an old animal. GFAP corresponding to astrocyte coverage is shown in green, p16 labeling indicating senescent cells is red; and DAPI illustrates the cell nuclei in blue. **(B)** Relative area covered by astrocytes labeled with GFAP in different forebrain regions in young and old animals. **(C)** The number of senescent astrocytes in young and old animals in different brain regions. Individual data points represent the mean value of three sections in each animal. Bar charts show mean ± stdev. The normal distribution of data was evaluated with a Shapiro–Wilk test (**B**: *p* = 0.105, **C**: *p* < 0.050-failed). For statistical analysis repeated measures model was used (RM ANOVA), followed by the Holm Sidak *post hoc* test. Levels of significance were set as *p* < 0.05^*^, *p* < 0.01^**^, *p* < 0.001^***^ vs. Young vehicle.

In aged animals, we found a significantly higher relative area covered by GFAP-labelled astrocytes in most brain regions examined (dentate gyrus: 10.9 ± 4.6 vs. 4.1 ± 2.1%; hippocampal CA1: 10.4 ± 4.6 vs. 4.8 ± 0.9%, Old control vs. Young vehicle) (Figure 5B).

In addition, aging had a profound effect on astrocyte senescence. The number of senescent astrocytes increased significantly in the old group compared to the young in most of the examined areas (Figure 5C), in the caudal part of the dentate gyrus (13.4 ± 4.8 vs. 3.9 ± 1.7 p16+ astrocytes/mm^2^ Old control vs. Young vehicle), the caudal hippocampal CA1 region (6 mm to bregma: 11.3 ± 2.5 vs. 5.3 ± 2.4 p16+ astrocytes/mm^2^, Old control vs. Young vehicle) as well as in the striatum (12.2 ± 3.5 vs. 4.0 ± 0.9 p16+ astrocytes/mm^2^, Old control vs. Young vehicle), and the dentate gyrus (13.2 ± 4.1 vs. 5.1 ± 2.5 p16+ astrocytes/mm^2^, Old control vs. Young vehicle). Increased number of senescent astrocytes was counted in the hippocampal CA1 region, as well (3 mm to bregma: 12.6 ± 4.4 vs. 3.4 ± 2.4 p16+ astrocytes/mm^2^, Old control vs. Young vehicle).

### 3.4. Effect of nimodipine on the age-related impairment of cerebrovascular reactivity

**Figure 6:**
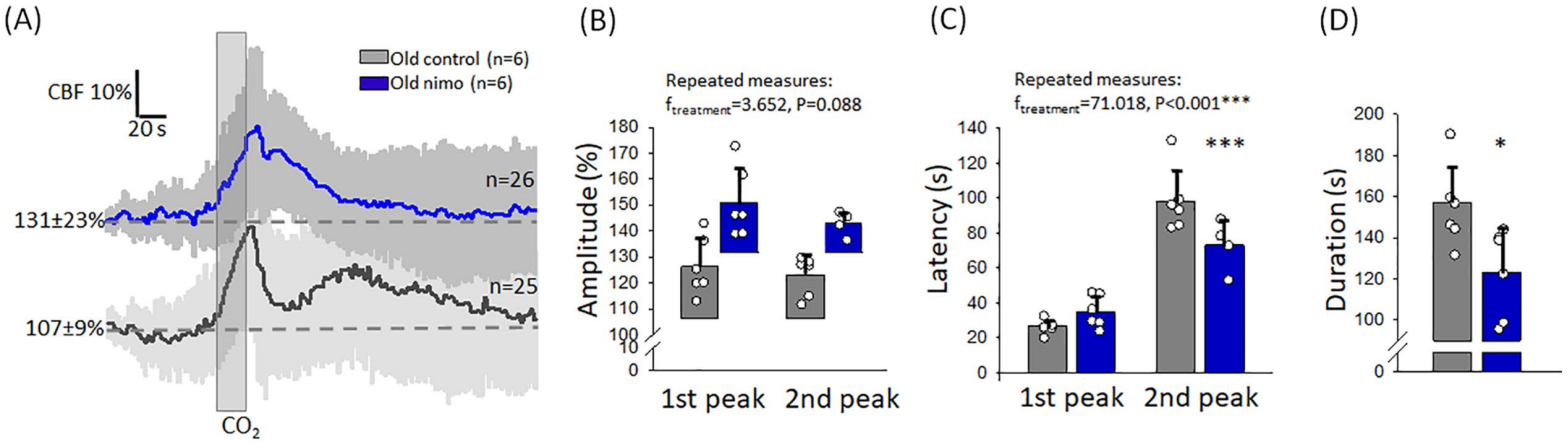
The impact of nimodipine treatment on the hyperemic response to hypercapnia in old animals. **(A)** Mean traces (±stdev) of the CBF response to hypercapnia in the Old control and nimodipine-treated Old nimo groups. **(B)** The amplitude of hyperemia. Note that the bars showing the relative amplitude are positioned on the CBF value preceding hypercapnia, to reflect the absolute amplitude of the CBF response. **(C)** The latency of the peak of hyperemia with respect to the onset of hypercapnia. **(D)** The duration of hyperemia. In **B-D**, data are given as mean ± stdev. The normal distribution of data was evaluated with a Shapiro–Wilk test (**B**: *p* = 0.124-failed, **C**: *p* = 0.125, **D**: *p* = 0.999). In **B-C**, RM ANOVA was used, followed by a Holm Sidak *post hoc* test. In **D**, one-way ANOVA was applied, followed by a Holm Sidak *post hoc* test. Levels of significance are labeled as *p* < 0.05^*^, *p* < 0.001^***^, vs. Old control.

Nimodipine treatment improved CVR impaired by age (Figure 6). First, the kinetics of the CBF response has been altered after nimodipine treatment. The second, blunted peak typical of the Old control group appeared to merge with the first, sharp peak, and the shape of the response has become more compatible with the monophasic response seen in young rats. This was confirmed by the shorter latency of the second peak still discernible (73 ± 15 vs. 98 ± 18 s, Old nimo vs. Old control) (Figure 6C), and the shorter duration of the CBF response (123 ± 21 vs. 156 ± 18 s, Old nimo vs. Old control) (Figure 6D). Although the statistical analysis did not show significant increase in the amplitude of hyperemia after nimodipine treatment, an upward tendency (*p* < 0.09) was observed in the absolute amplitude (1st peak: 150 ± 36 vs. 126 ± 15%, 2nd peak: 133 ± 23 vs. 124 ± 14%, Old nimo vs. Old control) (Figure 6B).

### 3.5. Effect of nimodipine on the acute cerebral ischemia-related impairment of cerebrovascular reactivity

**Figure 7:**
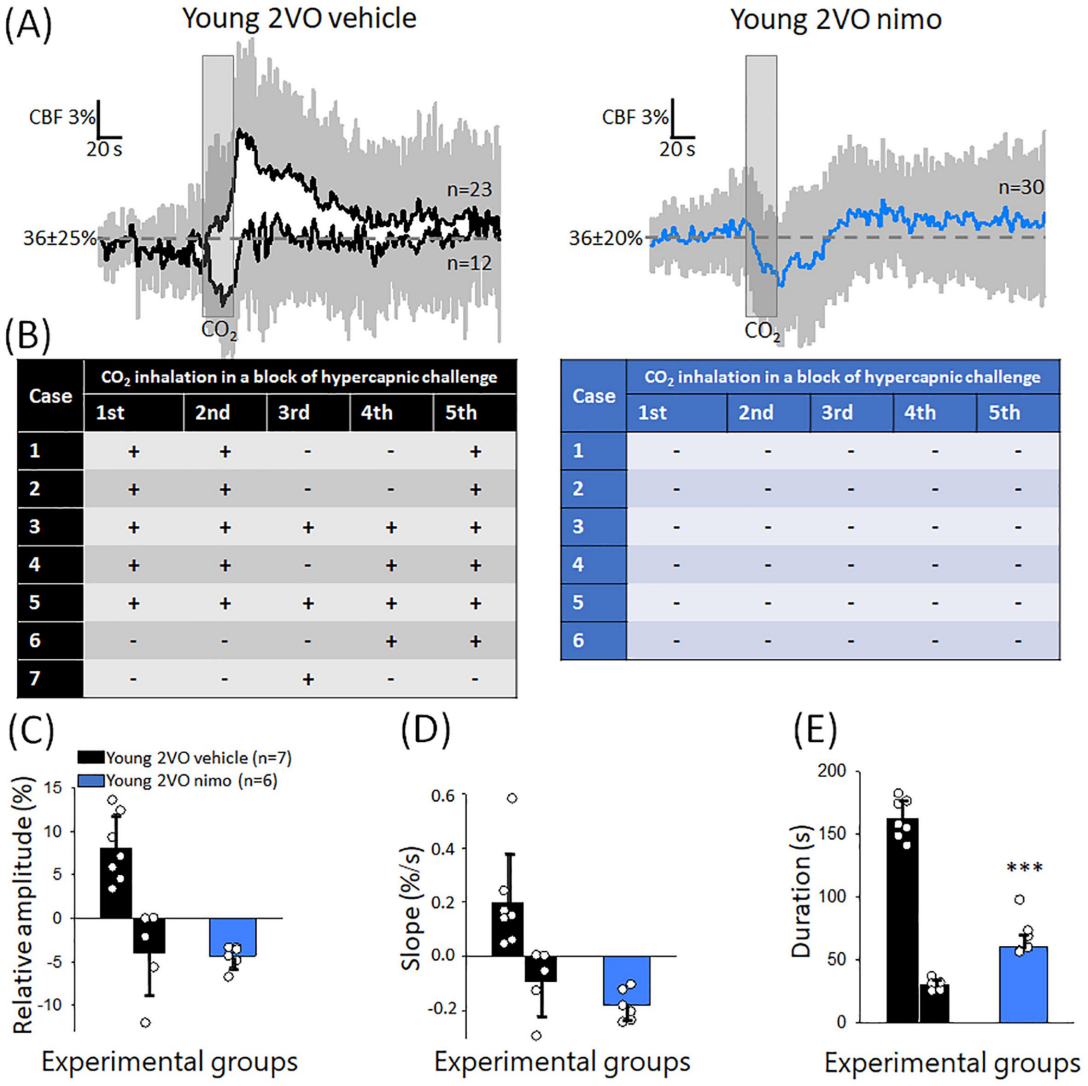
The effect of nimodipine treatment on the CBF response to hypercapnia during the acute phase of cerebral ischemia. **(A)** Mean traces (±stdev) of the CBF response to hypercapnia in Young 2VO vehicle and Young 2VO nimo animals. **(B)** Tables provide a summary of the occurrence of negative or positive CBF deflections with each CO_2_ inhalation trial in a block of hypercapnic challenge in the Young 2VO vehicle group (black) and the Young 2VO nimo group (blue). **(C)** The relative amplitude of CBF change. **(D)** The rate of hyperemia evolution (rising slope). **(E)** The duration of the CBF response. In **C-E**, data are given as mean ± stdev, and the means of positive and negative CBF responses are plotted apart (distinct bars) in the Young 2VO vehicle group. For statistical comparison in **C-D**, only the matching, negative values were taken (second bar in the black bar pairs). The normal distribution of data was evaluated with a Shapiro–Wilk test (**C**: *p* = 0.125, **D**: *p* = <0.228 **E**: *p* = 0.513). One-way ANOVA was then applied, followed by a Holm Sidak *post hoc* test. Level of significance is shown as *p* < 0.001^***^ vs. Young 2VO vehicle, inverse CBF response.

In contrast with the beneficial effect of nimodipine seen in the old animals, nimodipine treatment aggravated the impairment of CVR during the acute phase of cerebral ischemia. This manifested in an inverse CBF response to hypercapnia in all trials in all the animals (Figures 7A,B). CBF reduction rather than CBF elevation was noted in association with the hypercapnic challenge. Accordingly, the relative amplitude of the CBF response fell below pre-stimulation level (−4.3 ± 1.3%) (Figure 7C). The transient CBF reduction with hypercapnia after nimodipine application evolved similar to that seen in some animals that received vehicle (rate of CBF reduction: −4.3 ± 1.3 and − 3.8 ± 4.9%, Young 2VO nimo and Young 2VO vehicle Figure 7D), but the duration of the CBF drop was twice as long after nimodipine treatment (60.6 ± 8.8 vs. 30.3 ± 4.8 s, Young 2VO nimo vs. Young 2VO vehicle) (Figure 7E).

## 4. Discussion

The main objective of the present study was to investigate the possible protective effect of nimodipine on CVR gradually impaired during aging and abruptly disrupted after ischemia onset. The use of nimodipine, a dihydropyridine L-type VGCC blocker and cerebral vasodilator, was proposed to improve memory and sensorimotor function deteriorating with aging and to be beneficial to alleviate neurological deficit in ischemic stroke (Gelmers et al., 1988; Scriabine et al., 1989; Gelmers and Hennerici, 1990; Carlson et al., 2020). Although nimodipine’s hemodynamic side effects contraindicated its general use in ischemic stroke care (Wahlgren et al., 1994), nimodipine administration has been approved to prevent delayed ischemic deficit after subarachnoid hemorrhage (Tomassoni et al., 2008).

### 4.1. CVR in the aging brain and the impact of nimodipine

Impaired CVR to hypercapnia has been accepted as an indicator of the risk of cerebrovascular disease (Juttukonda and Donahue, 2019). Clinical studies addressing the impact of age on CVR demonstrated lower hypercapnic vasodilation of healthy aging compared to young individuals, by using transcranial Doppler ultrasound or MRI (Lu et al., 2011; Flück et al., 2014; Miller et al., 2019; Koep et al., 2022; Yew et al., 2022). Others reported preserved CVR in aging adults (Kastrup et al., 1998; Galvin et al., 2010; Stefanidis et al., 2019). Experimental studies found impaired pial arteriolar dilation in response to hypercapnia but no reduction of the regional CBF response in 8–12 months old mice compared to 6-week-old young animals (Balbi et al., 2015). On the other hand, perfusion maps generated by MRI disclosed significantly smaller perfusion increase in response to hypercapnia in 28.5 months old rats compared to their 12 months old adult counterparts (Mitschelen et al., 2009).

Here we report that the CBF increase to hypercapnia in the cerebral cortex is significantly smaller in 18–20 months old compared to 2 months old rats, which is consistent with previous experimental results (Mitschelen et al., 2009). The smaller CVR to hypercapnia may be attributed to the rarefaction of the cerebrovascular network with aging (Sonntag et al., 1997; Riddle et al., 2003; Faber et al., 2011). However, evidence for cerebrovascular rarefaction concerns the very late phase of life in rodents; indeed, reduced pial vascular density was seen in 30 months old rats (Sonntag et al., 1997) and 31 months old mice (Faber et al., 2011). In the age group studied here (18–20 months), we previously observed no rarefaction in the pial and cerebrocortical penetrating vasculature in rats (Bálint et al., 2019). Consistent with our earlier data, the density of the vascular SMC marker αSMA was similar in young and old rats in the current experiments. We, therefore, hypothesized that the senescence of cerebrovascular SMCs that execute vasodilation and relax in response to extracellular pH reduction (Apkon et al., 1997) must have contributed to the age-related CVR deficit. The senescence of vascular SMCs is thought to affect their responsiveness to contracting and relaxing stimuli, and the communication of vascular SMCs with the extracellular matrix (Chi et al., 2019). Our data reveal that cerebrovascular SMC senescence is obvious at the age of 18 months in rats and is coincident with the reduced efficacy of CVR.

Astrocyte senescence has also been considered as a potential contributor to the age-related CVR deficit. The assumption was based on the finding that hypercapnia caused intracellular Ca^2+^ surge and COX-1 activation in astrocytes, which led to vasodilator PGE_2_ production to suggest a regulatory role of astrocytes in hypercapnic vasodilation (Howarth et al., 2017). We have found an age-related increase of GFAP-positive astrocytes in the hippocampus CA1 and dentate gyrus, which is consistent with the progressive hypertrophy of astrocytes with increasing age shown earlier (Rodríguez et al., 2014). Most importantly, in addition to astrocyte hypertrophy, we have shown significant astrocyte senescence in our samples from old animals, which was coincident with CVR impairment. Taken together, our data demonstrate that CVR to hypercapnia becomes dysfunctional in aging rats, and the age-related CVR deficit coincides with cerebrovascular SMC and astrocyte senescence.

Nimodipine was reported to weaken CVR in early studies (McCalden et al., 1984; Schmidt et al., 1991), but a more recent NIRS examination of healthy adult volunteers found that nimodipine improved CVR to hypercapnia (Canova et al., 2012). Consistent with these later results, the administration of nimodipine here restored the morphology of the CBF response to that seen in young animals and showed a tendency (*p* = 0.088) to elevate the absolute peak of hypercapnic vasodilation in old rats. The beneficial effect of nimodipine on CVR is complemented by our recent finding that nimodipine enlarges functional hyperemia to somatosensory stimulation in the wake of spreading depolarization in the otherwise intact cortex (Menyhárt et al., 2023). The improvement in CVR weakened by aging may have functional benefits. In aged rats, CVR to hypercapnia was found particularly dysfunctional in a subpopulation of rats that was cognitively impaired (Mitschelen et al., 2009). Later clinical observations revealed that CVR efficacy in the hippocampus and temporal lobe correlated with memory performance in healthy older adults (Catchlove et al., 2018). The pharmacological improvement of CVR as seen here, therefore, may yield benefits for cognition and memory in aging.

### 4.2. CVR during acute cerebral ischemia and the impact of nimodipine

CVR impairment revealed by hypercapnic challenges emerges as a frequent consequence of acute and chronic cerebral ischemia (Leffler et al., 1989a,b; Dettmers et al., 1993; Ringelstein et al., 1994; Bari et al., 1998; Pfefferkorn et al., 2001; Conklin et al., 2010). Further, the degree of CVR deficit appears to predict stroke occurrence in patients with severe carotid or intracranial artery stenosis (Reinhard et al., 2014; Papassin et al., 2021) and may also reflect the severity of injury in ischemic stroke (Dettmers et al., 1993). We have observed in our global forebrain ischemia model that the hypercapnic CBF increase was profoundly impaired half an hour after ischemia onset. The impairment manifested in greatly reduced hyperemia, or a paradoxical negative CBF response to a hypercapnic challenge. The form of impairment (a small positive or negative CBF deflection) must have corresponded with the degree of perfusion deficit in the cortical region monitored, because in focal cerebral ischemia, a negative response to CO_2_ inhalation was associated with the core region, both negative and positive CBF deflections were seen in the penumbra, and the CBF response was predominantly positive more distant to the developing infarct (Dettmers et al., 1993). In our experiments, the perfusion deficit at the time of the hypercapnic challenge ranged between 20–40% of baseline, which represented a penumbra-like condition (Astrup et al., 1977).

The negative CBF response to hypercapnia seen here appears to be similar to the reversal of pial arteriolar dilation to vasoconstriction with CO_2_ inhalation after transient cerebral ischemia, described earlier in newborn piglets (Leffler et al., 1989a,b). Hypercapnia in the piglet model significantly increased the concentration of vasoactive prostanoids in the peri-arachnoid CSF in the intact brain. However, prostanoid concentrations did not increase with hypercapnia after an ischemic episode (Leffler et al., 1989a). This is in agreement with the recent finding that astrocytic COX-1 activation contributes to hypercapnic cerebral vasodilation (Howarth et al., 2017). The defect in vasoactive prostanoid synthesis, therefore, may account for the inverted CBF responses we have observed after ischemia onset.

The administration of nimodipine during the acute phase of cerebral ischemia unexpectedly increased the frequency of the negative CBF response type to hypercapnia. In fact, the CBF response was negative to each trial, and lasted significantly longer than in the untreated group. The negative CBF response to hypercapnia in the nimodipine-treated animals is interpreted as an intracerebral steal effect (Oshima et al., 2000; Venkatraghavan et al., 2018). The steal phenomenon occurs when blood is redistributed from regions of exhausted cerebrovascular reserve to areas with preserved vasodilator capacity in response to a vasodilator stimulus (Symon, 1969). Nimodipine is a vasodilator compound, therefore it appears plausible that nimodipine application augmented myogenic vasodilation typically occurring in response to reduced perfusion pressure after vascular occlusion (Palomares and Cipolla, 2011). Vasorelaxation after nimodipine application is thought to have reached a ceiling. Because nimodipine was applied topically, the suggested effect must have primarily concerned the region being treated and monitored. Consequently, cerebrovascular reserve at the site of interest was locally exhausted. The subsequent, global hypercapnic challenge then must have caused cerebral vasodilation distant to the cortical site under investigation and produced steal at the area treated with nimodipine.

Clinical and experimental studies did not find evidence that nimodipine might cause intracerebral steal in ischemic stroke (Gelmers, 1982; Date and Hossmann, 1984). However, the Intravenous Nimodipine West European Stroke Trial warned that the hemodynamic side effects – with blood pressure reduction in the focus – might outweigh the neuroprotective benefit of nimodipine (Wahlgren et al., 1994). Along this line, the potentiation of the steal phenomenon by nimodipine as seen here in the acute phase of cerebral ischemia prompts further investigation, and must be considered as a possible undesirable side effect of the drug. Indeed, the steal phenomenon carries the risk of progressive tissue injury (Mandell et al., 2008; Kim et al., 2012).

### 4.3. Limitations

It remains a translational limitation of the current experimental approach that nimodipine was applied in the hyperacute phase after vascular occlusion, very early after ischemia onset. However, the ischemic penumbra of a focal infarct evolves over space and time, and our observations made concerning penumbra conditions may be applicable for penumbra tissue at later time points, as well. It may also be perceived as a limitation that the experiments were terminated without evaluating functional outcomes and thus cerebroprotection (Lyden et al., 2021). The invasive nature of the surgical procedures (i.e., craniotomy) in our experiments justified the termination of the experiments without the discontinuation of anesthesia. Lastly, the current research addressed aging by studying an old group of rats but did not tackle sex differences, because extensive previous work has not suggested any potential sex-related differences in responsiveness to nimodipine (Wadworth and McTavish, 1992; Carlson et al., 2020). The interpretation of the present research results must take these limitations into consideration.

### 4.4. Nimodipine: risks and benefits

Nimodipine, a drug approved by the FDA in 1988 for the improvement of neurologic outcomes after aneurysmal subarachnoid hemorrhage was also considered for the treatment of ischemic stroke and age-related cognitive disorders (Carlson et al., 2020). In ischemic stroke, after initial encouraging observations, clinical trials terminated because detrimental side effects, especially hypotension and the related aggravation of neural injury contraindicated the use of nimodipine. Nimodipine application to alleviate cognitive impairment was also abandoned because of short-term and moderate benefits. Since then, major advancements have been made in two fields. First, pharmaceutical technology developed various approaches for controlled, targeted drug delivery to improve drug effectiveness and limit adverse side effects (Carlson et al., 2020; Tóth et al., 2020a). Second, additional pathomechanisms of neuronal injury (e.g., spreading depolarization) have been recognized as novel targets for nimodipine (Carlson et al., 2020). The use of nimodipine as a cerebroprotective substance may, therefore, be worth revisiting.

The current experimental work draws attention to potential risks and benefits of nimodipine. In our model of healthy aging, nimodipine offered cerebrovascular benefit, while in the acute global cerebral ischemia model, the cerebrovascular effects of nimodipine to a systemic physiological challenge proved to be disadvantageous. Based on these results, one may speculate that the risks of nimodipine administration increase with the severity of the cerebral metabolic stress. It is beyond the current experimental work to argue for or against the use of nimodipine for the treatment of ischemic stroke, but these data may prompt further investigation to evaluate the balance between risks and benefits of nimodipine administration under various cerebral metabolic states.

## 5. Conclusion

We demonstrate here that CVR becomes inefficient with aging coincident with cerebrovascular SMC and astrocyte senescence, and can be augmented by nimodipine. Further, CVR becomes severely impaired in acute ischemia, but nimodipine administration in the acute phase to rescue ischemia-related CVR deficit carries the risk of the steal effect. Nimodipine administration may offer benefits in aging and age-related cognitive dysfunction by improving CVR. However, a careful evaluation of expected benefits and potential side effects of nimodipine treatment is warranted is acute ischemic stroke.

## Data availability statement

The raw data supporting the conclusions of this article will be made available by the authors, without undue reservation.

## Ethics statement

The animal study was reviewed and approved by the National Food Chain Safety and Animal Health Directorate of Csongrád County, Hungary.

## Author contributions

SK: conceptualization, data curation, formal analysis, and writing–original draft. ÁM: conceptualization, methodology, supervision, and writing–review and editing. FB: funding acquisition and writing–review and editing. EF: conceptualization, formal analysis, funding acquisition, and writing–original draft. All authors contributed to the article and approved the submitted version.

## Funding

The EU’s Horizon 2020 research and innovation program no. 739593; grants from the National Research, Development and Innovation Office of Hungary (nos. K134377, K134334 and FK142218); the Ministry of Innovation and Technology of Hungary and the National Research, Development and Innovation Fund (no. TKP2021-EGA-28 financed under the TKP2021-EGA funding scheme); the National Brain Research Program 3.0 of the Hungarian Academy of Sciences; and the Research Fund of the Albert Szent-Györgyi Medical School, University of Szeged, Hungary.

## Conflict of interest

The authors declare that the research was conducted in the absence of any commercial or financial relationships that could be construed as a potential conflict of interest.

## Publisher’s note

All claims expressed in this article are solely those of the authors and do not necessarily represent those of their affiliated organizations, or those of the publisher, the editors and the reviewers. Any product that may be evaluated in this article, or claim that may be made by its manufacturer, is not guaranteed or endorsed by the publisher.

## Figures and Tables

**Table 1:** 

2VO	bilateral occlusion of the common carotid arteries (two-vessel occlusion)
αSMA	alpha smooth muscle actin
aCSF	artificial cerebrospinal fluid
ANOVA	analysis of Variance
CBF	cerebral blood flow
COX-1	cyclooxygenase-1
CVR	cerebrovascular reactivity
GFAP	glial fibrillar acidic protein
LDF	laser-Doppler flowmetry
MABP	mean arterial blood pressure
NGS	normal goat serum
PBS	phosphate buffered saline
PFA	paraformaldehyde
PFU	perfusion units
PGE_2_	prostaglandin E_2_
SMC	smooth muscle cell
VGCC	voltage-gated calcium channel
